# Perceived Confidence in Managing Medical Emergencies Among Dental Students: The Significance of Technical Knowledge and Emotional Intelligence

**DOI:** 10.1111/eje.70151

**Published:** 2026-04-01

**Authors:** Tecianny Nunes de Aquino, Taysllan Torquato Benevides, Maria Luísa de Assis Braga, Annyelle Anastácio Cordeiro, Ana Flávia Granville‐Garcia, Érick Tássio Barbosa Neves

**Affiliations:** ^1^ School of Dentistry Universidade Estadual da Paraíba – UEPB Araruna Paraíba Brazil; ^2^ School of Dentistry Universidade Estadual da Paraíba – UEPB Campina Grande Paraíba Brazil

**Keywords:** confidence, dental, dentistry, emergency treatment, emotional intelligence, students

## Abstract

**Aim:**

This study aimed to evaluate the technical, practical and emotional factors associated with the self‐reported confidence of dental students in managing medical emergencies and urgent situations.

**Materials and Methods:**

A cross‐sectional study was conducted with 100 students enrolled in the clinical courses of a public dentistry programme in Brazil. Participants completed a sociodemographic questionnaire, an instrument assessing preparation and experience with medical emergencies in dental practice, and the Trait Emotional Intelligence Questionnaire–Short Form (TEIQue‐SF), validated for use in Brazil. Statistical analyses were performed using SPSS v.25.0, applying frequency distributions, Fisher's exact test and Student's *t*‐test, with significance set at *p* < 0.05.

**Results:**

Most students reported not feeling prepared to manage emergencies (82.0%), diagnose complications (61.0%) or administer injectable medications (91.0%). The mean emotional intelligence score was 5.06 ± 0.49. Perceived confidence was significantly higher among students who reported satisfactory technical and practical training, preparedness to diagnose emergencies and ability to administer injectable medications (*p* < 0.01). Students who considered themselves prepared to make diagnoses also demonstrated significantly higher emotional intelligence scores (*p* = 0.04).

**Conclusions:**

Dental students showed low perceived confidence in managing medical emergencies, a perception influenced by technical training, practical training and emotional intelligence. This study provides insight into determinants influencing dental students' confidence in managing medical emergencies, highlighting training and emotional intelligence as key factors.

## 1 Introduction

Medical emergencies and urgent situations in dental practice are relatively common, with estimates indicating at least one case for every one to two years of clinical practice per dentist [1]. Reported prevalence in the literature varies widely, from 0.9% to 66.9% [2, 3]. The most frequent episodes include vasovagal syncope, hypoglycemia and allergic reactions, whereas rarer but potentially life‐threatening events include anaphylactic shock, hypertensive crises, angina pectoris and cardiopulmonary arrest [4]. Because of the potential for such complications, dentists must remain up to date and demonstrate both technical knowledge and practical skills to ensure a rapid, effective and safe response [5].

In this regard, self‐reported confidence in handling medical emergencies represents a critical component of clinical practice, as it directly influences decision‐making in high‐stakes situations [6]. Confidence begins to develop during academic training and is shaped by theoretical and practical experiences, the ability to administer injectable medications in emergency contexts, and the capacity to diagnose and appropriately manage different clinical situations [7].

A key factor recognised for its role in shaping behaviour is emotional intelligence (EI), defined as the ability to recognise and regulate personal emotions, as well as to perceive and influence the emotions of others [8]. In healthcare, EI serves as an important behavioural regulator, promoting more effective, safe and ethical clinical conduct [9]. Although recognition of EI within the health sciences has grown, no studies have specifically examined its influence on dental students and their perceived confidence when managing urgent and emergent medical situations. Assessing the relationship between EI and perceived confidence in managing emergencies in dentistry is therefore both relevant and innovative, as it may broaden understanding of the factors that shape clinical performance in emotionally demanding contexts.

The conceptual hypothesis of this study is that both technical knowledge related to the recognition and management of medical emergencies and EI directly influence dental students and their self‐reported sense of confidence in such situations during clinical practice. Accordingly, the aim of this study is to analyse emotional, theoretical and practical factors associated with self‐reported confidence in managing medical complications in clinical practice among dental students.

## 2 Materials and Methods

This study was conducted in accordance with the ethical principles of the Declaration of Helsinki and received approval from the Human Research Ethics Committee of the State University of Paraiba (protocol: 82491024.8.0000.5187).

### 2.1 Design and Sampling Procedure

This observational, cross‐sectional, analytical study was carried out at the State University of Paraiba, with students enrolled in the undergraduate Dentistry programme. The programme is structured around laboratories and clinics that provide students with comprehensive, integrated training while addressing the clinical needs of the local population and surrounding communities. Through these services, the community receives access to a wide range of dental care, from basic procedures such as restorations and scaling to more complex interventions, including periodontal surgery, tooth extractions, frenectomy and laser therapy. A census‐based sampling strategy was employed, including all students enrolled in the clinical axis of the undergraduate programme, yielding a final sample of 100 participants. Post hoc statistical power for the main analyses was calculated using G*Power software (version 3.1.9.7).

### 2.2 Eligibility Criteria

All students aged 18 years or older who were enrolled in the clinical courses of the undergraduate Dentistry programme (fifth to tenth semesters) during the data collection period were eligible for inclusion. Students were excluded if they had prior academic training in medical emergencies from other health‐related fields or if they were active rescue workers.

### 2.3 Data Collection and Pilot Study

Data collection took place between April and June 2025 and involved the administration of questionnaires to gather sociodemographic information, evaluate self‐reported confidence in managing medical emergencies and urgent situations in dentistry, and assess levels of EI. A trained researcher administered the questionnaires to the participating students.

Socioeconomic and demographic information was obtained using a structured questionnaire that included variables such as age, sex, family income, self‐declared race and current semester. Students then answered both objective and open‐ended questions regarding their knowledge, practical experience and educational background related to medical emergencies in dentistry. Perceived confidence in handling such situations was assessed with the following question: ‘Do you feel confident dealing with medical emergencies and urgent situations in dental practice?’

EI was measured using the Trait Emotional Intelligence Questionnaire–Short Form (TEIQue‐SF), a validated instrument in Brazil, consisting of 30 items rated on a 7‐point Likert scale ranging from ‘strongly disagree’ (1) to ‘strongly agree’ (7). The TEIQue‐SF adopts a psychological perspective and evaluates both positive and negative aspects of psychological states, including well‐being, absence of unpleasant symptoms, self‐confidence, health‐related locus of control, optimism, life satisfaction and emotional responses to diverse situations. The total EI score was calculated as the arithmetic mean of the 30 items, following the scoring guidelines proposed by the instrument developers. Higher scores indicate higher trait emotional intelligence. The Brazilian validation study demonstrated adequate internal consistency (α = 0.88) and satisfactory psychometric properties [10]. Students were invited to participate after leaving the teaching clinics and were instructed to complete the questionnaire individually, in a private setting, without interference from the administrator.

### 2.4 Statistical Analysis

Statistical analyses were performed using SPSS Statistics version 25.0. Categorical variables were expressed as absolute and relative frequencies, and associations were tested using Fisher's exact test. A significance level of 5% (α = 0.05) was adopted. Quantitative variables were described as means and standard deviations, and group comparisons were conducted using Student's *t*‐test for independent samples after verification of normality. Analyses focused on examining associations between sociodemographic and educational characteristics and self‐reported confidence in managing medical emergencies in dentistry, as well as potential associations with EI.

With a sample of 100 participants, statistical power was estimated for mean comparisons and differences between proportions corresponding to the applied tests. Assuming a moderate effect size (d = 0.4) and a significance level of 5% (α = 0.05), statistical power exceeded 0.80, indicating that the sample was sufficient to detect statistically significant effects.

## 3 Results

The final sample consisted of 100 dental students, yielding a response rate of 94.0%. Losses resulted from three consecutive absences during the data collection phase. The mean age was 22.8 ± 2.97 years, with a predominance of women (68.0%) and self‐declared non‐white participants.

In relation to the management of medical emergencies in dental practice, 61.0% of participants reported feeling insecure about making a diagnosis and 82.0% about managing such situations. Furthermore, 91.0% reported insecurity in administering injectable medications, and half rated their theoretical and practical experience in these situations as unsatisfactory. For EI, assessed with the TEIQue‐SF, the mean score was 5.06 ± 0.49. Table 1 presents the detailed characterisation of the sample.

**TABLE 1:** Sociodemographic characteristics and perceived confidence in managing urgent/emergency situations among dental students.

Variable	Frequency	
	*n*	%
Sex		
Male	32	32.0
Female	68	68.0
Semester of study		
5th–7th (beginning of clinical cycle)	54	54.0
8th–10th (end of clinical cycle)	46	46.0
Prepared to diagnose U.E.^a^		
No	61	61.0
Yes	39	39.0
Feels confident in managing U.E.^a^		
No	82	82.0
Yes	18	18.0
Ability to administer injectable medications		
No	90	91.0
Yes	9	9.0
Theoretical and practical experience in U.E.^a^		
Unsatisfactory	50	50.0
Regular/Satisfactory	50	50.0

^a^ U.E. = Urgent and Emergency situations.

Table 2 presents the factors associated with self‐reported confidence in urgent and emergency situations among dental students. A greater perception of confidence was observed among those who reported regular or satisfactory academic experience, indicated that they knew how to diagnose such situations, and reported ability to administer injectable medications (*p* < 0.01).

**TABLE 2:** Factors associated with self‐reported confidence in urgent and emergency situations among dental students.

Variable	Confident	Not confident	*p*
Theoretical and practical experience in U.E.^a^			< 0.01
Unsatisfactory	3 (6.0%)	47 (94.0%)	
Regular/Satisfactory	15 (30.0%)	35 (70.0%)	
Ability to administer injectable medications			< 0.01
No	13 (14.3%)	78 (85.7%)	
Yes	5 (55.6%)	4 (44.4%)	
Prepared to diagnose U.E.^a^			< 0.01
No	0 (0.0%)	61 (100.0%)	
Yes	18 (46.2%)	21 (53.8%)	
Sex			0.09
Male	9 (28.1%)	23 (71.9%)	
Female	9 (13.2%)	59 (86.8%)	

*Note:* Fisher's exact test.

^a^ U.E. = urgent and emergency situations.

Table 3 presents the association between levels of EI and factors related to the management of urgent and emergency situations in the dental clinic. Participants with higher EI scores (5.21 ± 0.56) were more likely to consider themselves prepared to diagnose emergencies compared with those with lower scores (5.00 ± 0.44; *p* = 0.04). Higher EI also tended to be observed among students with more satisfactory theoretical and practical experience (5.13 ± 0.52), those who reported ability to administer injectable medications (5.11 ± 0.33), and women (5.14 ± 0.50), although these differences did not reach statistical significance.

**TABLE 3:** Association between factors related to the management of urgent and emergency situations in dental clinics and emotional intelligence among dental students.

Variable	Mean EI^c^ (M^a^ ± SD^b^)	*p*
Theoretical and practical experience in U.E.^d^		0.348
Unsatisfactory	5.04 ± 0.48	
Regular/Satisfactory	5.13 ± 0.52	
Ability to administer injectable medications		0.845
No	5.08 ± 0.51	
Yes	5.11 ± 0.33	
Prepared to diagnose U.E.^d^		0.040
No	5.00 ± 0.44	
Yes	5.21 ± 0.56	
Feels confident in managing U.E.^d^		0.740
No	5.09 ± 0.50	
Yes	5.05 ± 0.51	
Sex		0.411
Female	5.14 ± 0.50	
Male	5.06 ± 0.50	

*Note:* Student's *t*‐test.

^a^ M = mean.

^b^ SD = standard deviation.

^c^ E.I. = emotional intelligence.

^d^ U.E = urgent and emergency situations.

## 4 Discussion

The present study demonstrated that self‐reported theoretical and practical experience, along with EI, are factors related to the perceived confidence of dental students in managing medical emergencies and urgent situations. The results indicated a low perception of preparedness, corroborating previous findings that highlight insufficient dental training for these scenarios [3, 5]. This situation is concerning, given that the public assumes that dentists possess the necessary qualifications to handle medical emergencies [11]. Such trust, reinforced by the requirements of the Dental Code of Ethics and current legislation, emphasises professional responsibility and establishes an ethical and legal obligation to ensure confidence and competence in patient care.

The prevalence of students who considered themselves unable to safely diagnose and manage medical emergencies and urgent situations in dentistry was high in this study (82.0%). Similar results have been reported in national and international investigations (78.9%–92.1%), suggesting limitations in student training and highlighting the need to strengthen teaching strategies in dentistry curricula [12, 13].

Regarding theoretical and practical experience with medical emergencies in dental practice, students who reported unsatisfactory experience demonstrated lower confidence in managing such cases. This finding reinforces the importance of targeted theoretical and practical training, which is essential for internalising protocols and developing confidence in critical situations, emphasising the need for greater focus on practical training during undergraduate education [6, 7, 14].

Among the practical skills essential for managing medical emergencies and urgent situations, a self‐reported lack of knowledge about administering injectable medications was associated with lower confidence in handling such cases. Previous studies have also documented that dentists report low confidence in administering injectable medications [15]. This finding highlights a critical gap in dental education, as several clinical emergencies, including severe allergic reactions, hypoglycemia and seizures, require the initial use of parenteral medications [4].

With regard to EI, this appears to be the first study to assess its impact on factors related to the management of urgent and emergency situations in dental clinics by students. Higher EI scores were associated with a greater perception of preparedness to diagnose medical emergencies, suggesting that EI may positively influence self‐confidence in critical situations [16]. The role of EI in the academic success of dental students—mitigating negative feelings, enhancing organisational performance and strengthening leadership attitudes—has been previously reported [17]. Therefore, EI should be considered an important element to integrate into educational practices designed for this population.

A limitation of this study is the inability to establish causal relationships due to its cross‐sectional design. Additionally, perceived confidence was assessed through self‐report measures, which are inherently subjective and may be influenced by personal bias. However, the study specifically aimed to investigate perceived confidence rather than objective competence, and such subjectivity is intrinsic to the construct evaluated. Therefore, the findings should be interpreted cautiously. However, methodological precautions were implemented to enhance sample representativeness and study reproducibility, thereby strengthening internal and external validity. The findings highlight the need to reinforce the theoretical and practical approach to medical urgencies and emergencies in undergraduate dentistry, as well as to integrate basic life support training modules throughout the curriculum to improve knowledge, practical skills and attitudes among students.

## 5 Conclusion

Dental students showed low perceived confidence in managing medical emergencies, a perception influenced by theoretical training, practical training and emotional intelligence. This study provides insight into factors influencing dental students' perceived confidence in managing medical emergencies, highlighting the role of theoretical and practical training as well as emotional intelligence, and suggesting future educational strategies to enhance preparedness and confidence in clinical practice.

## Funding

This study was partially supported by an undergraduate scholarship from the Institutional Scientific Initiation Scholarship Program (CNPq), National Council for Scientific and Technological Development, Brazil.

## Conflicts of Interest

The authors declare no conflicts of interest.

## Data Availability

The data that support the findings of this study are available from the corresponding author upon reasonable request.
